# Temperature-Dependent Structural and Electrical Properties of Metal-Organic CVD MoS_2_ Films

**DOI:** 10.3390/nano13192712

**Published:** 2023-10-06

**Authors:** Roman I. Romanov, Ivan V. Zabrosaev, Anastasia A. Chouprik, Dmitry I. Yakubovsky, Mikhail K. Tatmyshevskiy, Valentyn S. Volkov, Andrey M. Markeev

**Affiliations:** 1 Center of Shared Research Facilities, Moscow Institute of Physics and Technology (National Research University), Dolgoprudny 141701, Russia; romanov.ri@mipt.ru (R.I.R.); zabrosaev@phystech.edu (I.V.Z.); chouprik.aa@mipt.ru (A.A.C.); 2 Center for Photonics & 2D Materials, Moscow Institute of Physics and Technology (National Research University), Dolgoprudny 141700, Russia; dmitrii.yakubovskii@phystech.edu (D.I.Y.); mikhail.tatmyshevskiy@phystech.edu (M.K.T.); volkov.vs@mipt.ru (V.S.V.)

**Keywords:** TMD, MOCVD, nanocrystalline film, AFM, Raman spectroscopy, XPS, TEM

## Abstract

Metal-Organic CVD method (MOCVD) allows for deposition of ultrathin 2D transition metal dichalcogenides (TMD) films of electronic quality onto wafer-scale substrates. In this work, the effect of temperature on structure, chemical states, and electronic qualities of the MOCVD MoS_2_ films were investigated. The results demonstrate that the temperature increase in the range of 650 °C to 950 °C results in non-monotonic average crystallite size variation. Atomic force microscopy (AFM), transmission electron microscopy (TEM), and Raman spectroscopy investigation has established the film crystal structure improvement with temperature increase in this range. At the same time, X-Ray photoelectron spectroscopy (XPS) method allowed to reveal non-stoichiometric phase fraction increase, corresponding to increased sulfur vacancies (V_S_) concentration from approximately 0.9 at.% to 3.6 at.%. Established dependency between the crystallite domains size and V_S_ concentration suggests that these vacancies are form predominantly at the grain boundaries. The results suggest that an increased Vs concentration and enhanced charge carriers scattering at the grains’ boundaries should be the primary reasons of films’ resistivity increase from 4 kΩ·cm to 39 kΩ·cm.

## 1. Introduction

Two-dimensional transition metal dichalcogenides (TMD) have been attracting increasing attention recently due to their unique properties and promising applications in such diverse areas as nanoelectronics [1,2], optoelectronics [3,4], data storage devices [5], photoelectric converters [6,7,8], gas sensors [9,10], energy storage devices [11,12] and catalysis [13,14]. As a FET channel material, they feature a tunable band gap in the suitable range and remain stable at thickness as low as a single monolayer. The use of such ultrathin channels helps mitigate the short channel effects and allows to obtain high channel electrostatic control [15,16]. Numerous successful applications, demonstrated on experimental samples, create the prerequisites for the widespread introduction of such materials in the new generation electronic devices, including phototransistors [17], ultrafast flash memory [18], volatile memory transistors [19]. Research efforts are currently focused on the transition from experimental devices based on single flakes to developing wafer-scale manufacturing technologies to meet production needs.

Among various film synthesis techniques the chemical vapor deposition (CVD) method [20,21,22], in particular metal-organic CVD (MOCVD) [23,24], as well as two-step sulfurization [25,26,27] methods are among the most widely used for uniform films synthesis over wafer scale substrates. The CVD method allows for electronic grade films fabrication. In addition, the transition from powder precursors to metalorganic and gaseous precursors makes it possible to significantly improve the controllability of the growth process, as well as the uniformity of the deposited films. However, the MOCVD method imposes certain limitations: crystalline size in such films is usually less than or on the order of a micrometer, the layer-by-layer growth regime has a narrow parameter window [24], and the possible presence of residual carbon phase in fabricated films [28]. In addition, numerous questions concerning the mechanisms of such film growth are still poorly investigated. In particular, the conditions for either physical or chemical adsorption [29], the effect of local carbon inclusions [30], etc.

The improvements of MOCVD synthesis method in recent years included the use of oxygen or water vapor as promoters [31,32,33], as well as reactor design improvements [33]. This made it possible to enhance the size of the crystallites and deposition uniformity. However, the investigation of the process parameters influence on resulting MoS_2_ film properties remains relevant. This includes the investigation of different growth regimes, allowing to vary the films’ structure and tune other parameters. This is particularly interesting due to the fact that in different areas of interest are TMD films with either polycrystalline or nanocrystalline and amorphous structure [34,35].

The significant focus of current research efforts is on the temperature effect on the MoS_2_ films growth [28,29,30], a wide temperature range of 400 °C to 1000 °C is investigated. The focus in these works was primarily on fabrication temperature effect on grain size. In general, it was found that higher deposition temperature results in grain size increase due to the nucleation density decrease. In [36] the substrate temperature varied from 620 °C to 845 °C. At 620 °C and 725 °C an early grain coalescence occurred, however grain size remained below 15 nm. At 845 °C the grains had triangle shape and their lateral size increased to 29–57 nm depending on the substrate pre-treatment. In [37] the temperature range of 400 °C to 600 °C was studied. It was shown that within this temperature range, the average grain size increased from about 10 nm to about 30 nm, with the lateral growth rate increasing from 40 nm/min to 100 nm/min. In another work [38] the effect of temperature in the range from 670 °C to 1070 °C was studied. With an increase in temperature to 785 °C, a nucleation density reduction was observed. With further temperature increase in the range from 785 °C to 905 °C, the nucleation density and the average size of crystallites did not change significantly. With an increase to 955 °C, the average crystallite size decreased. According to the authors, the range from 785 °C to 905 °C is optimal since it allows for better control over the process with varying precursor flows. In the work [39] the temperature range of 650–850 °C was focused. With the temperature increase from 650 °C to 750 °C, the average crystallite size increased from 500 nm^2^ to 2500 nm^2^. Within this temperature range, the authors identified three growth regimes: surface reactions limited (650–700 °C), mass transport limited (750–800 °C), and desorption limited (850 °C and above).

Raman spectroscopy is one of the most widely used methods for MoS_2_ crystal structure investigation. The MoS_2_ structure quality is estimated based on the E_2g_^1^ и A_1g_ peaks width, as well as specific peaks, corresponding to the lattice defects. Thus, in [36] it was found that with increasing temperature, the Full Width-Half Maximum (FWHM) of E_2g_^1^ peaks decreases from 4.7 ± 0.1 to 3.2 ± 0.3 (cm^−1^), and the FWHM of A_1g_ peaks decreases from 5.6 ± 0.1 to 4.5 ± 0.5 (cm^−1^). The XPS method is used to estimate the deviation of the film’s stoichiometry. Thus, using XPS in [37] the authors established that the S:Mo ratio depends on the process temperature, substrate material, and plasma power during substrate pretreatment.

Despite the fact that there are numerous works devoted to the investigation of temperature effect on MOCVD MoS_2_ films properties, the provided results do not sufficiently characterize the films. They focus primarily on the nucleation density and crystalline size. The effect on such factors as the elements’ chemical states, the defects concentration, grain boundaries structure, etc., as well as interconnections between these parameters have not been studied thoroughly. In this work, the optimal temperature range from 650 °C to 950 °C, as well as widely used precursors: molybdenum hexacarbonyl (Mo(CO)_6_) and hydrogen sulfide (H_2_S), were utilized. The goal of this work was a comprehensive study of the temperature effect on the structure and chemical states in MoS_2_ films including the states induced by the structural defects. Based on careful analysis of TEM, SEM, AFM, Raman spectroscopy and XPS data, we were able to establish the relationship between the grain boundaries density, determined by grain size, and the sulfur vacancies (V_S_) concentration. In addition, the influence of these factors on the electrical properties of the films was estimated.

## 2. Materials and Methods

The MOCVD process was conducted in a homemade reactor based on a three-zone tube furnace HZS-1200 (Carbolite Gero HZS 1200, Carbolite Gero, Hope Valley, UK) equipped with a 32 mm outer diameter quartz tube. Molybdenum hexacarbonyl (Mo(CO)_6_) and hydrogen sulfide (H_2_S) were used as the molybdenum and sulfur precursors, respectively. Ar + 5%H_2_ mixture was used as a carrier gas. Mo(CO)_6_ was kept in a stainless-steel bubbler with temperature control system, which allowed to sustain precursor temperature of 21.5 °C. The inlet gas flux rates of Mo(CO)_6_ (99.98% purity, Sigma-Aldrich, Burlington, MA, USA) were 3.1 × 10^−3^ sccm, inlet gas flow rate of H_2_S (99.9%) was 15 sccm for all samples. Carrier gas Ar + 5% H_2_ inlet flow rate was 600 sccm. Thus, the precursor flux ratio was approximately 4.8·10^3^. This parameter is very important as it determines the growth mechanism. At a low S:Mo precursor ratio, three-dimensional nuclear growth occurs. Variation of this parameter in the range that ensures two-dimensional growth regime affects the shape of crystalline domains and the V_S_ concentration [24]. The higher the growth temperature, the greater this ratio must be to compensate for sulfur desorption. However, in this work, the ratio of precursor flows was fixed in order to distinguish the temperature effect. The S:Mo ratio was chosen based on reported data to be high enough to ensure a two-dimensional growth regime and good stoichiometry for highest investigated temperature [40]. The detailed reactor scheme can be found in [41]. The reactor pressure was kept constant at 35 kPa, while the deposition temperature varied and was set to 650 °C, 750 °C, 850 °C, 950 °C. 20 × 10 mm-size c-plane sapphire pieces were used as substrates. They were piranha solution cleaned and subsequently washed in deionized water. They were further annealed in the same tube furnace which was used in CVD processes in the air for 1 h at 1000 °C. For CVD deposition samples were placed in the central section of the three-zone tube furnace. The tube was pumped down below 0.1 hPa prior to the process. The heating was performed in Ar + 5%H_2_ flow. The temperature increase rate was 30 °C/min. At the temperature setpoint, H_2_S flow was supplied, and after 10 min of stabilization time Mo(CO)_6_ precursor flow was supplied as well. At the end of CVD process, the Mo(CO)_6_ flow and furnace heaters were immediately switched off, while the H_2_S flow was maintained during the cooling stage down to 450 °C, below this temperature, only Ar + H_2_ mixture was supplied to the reactor. Process time for continuous film deposition varied from 8 to 9 h. Two series of samples were fabricated. The first one, at a decreased deposition time (4 to 6 h) to facilitate the grain size analysis. For the second series the deposition time was increased to 6 to 9 h in order to obtain continuous films. After the deposition process, the heating system was switched off, allowing the furnace to cool down.

The films’ surface was investigated using atomic force microscopy (AFM, NT-MDT N’tegra and Solver tools, Moscow, Russia) in a semi-contact mode using a silicon tip with a radius <10 nm (HA-NC, SCANSENS, Bremen, Germany). Scans were recorded over a 1 × 1 µm^2^ area for each film. Microstructural analysis was performed using a transmission electron microscope FEI Tecnai G2 (FEI Company, Hillsboro, OR, USA). For TEM analysis, MoS_2_ films were transferred without PMMA onto carbon-coated copper grids after exposure to the KOH solution. Raman spectroscopy was used to investigate the film molecular and crystal lattice structure. A LabRAM Evolution (Horiba Ltd., Kyoto, Japan) instrument with laser source of a 532 nm wavelength and 1 cm^−1^ spectral resolution was used for spectral measurements. A x100 objective lens with numerical aperture of 0.90 and the diffraction grating of 1800 lines/mm were utilized in the experiment. The laser spot diameter was 0.45 µm. In order to avoid measurement artifacts caused by heating a low power laser (0.5 mW) was used. All measurements were performed at standard conditions. The 520 cm^−1^ phonon mode of the silicon wafer was used for spectrometer calibration.

The chemical states of the elements and films composition were studied by XPS in the Theta Probe tool (Thermo Scientific, East Grinstead, UK) with a monochromatic Al-Kα X-ray source (1486.6 eV) in high-vacuum conditions. Photoelectron spectra were acquired using fixed analyzer transmission (FAT) mode with 50 eV pass energy. XPS spectra were acquired in charge-compensation mode under the pressure of ~10^−7^ mbar to avoid the influence of sapphire charging.

The films’ sheet resistance was estimated using four-probe method at the Keysight B1500A (Agilent Technologies, Tokyo, Japan) measurement unit and a Cascade Microtech Summit probe station (Cascade Microtech, Beaverton, OR, USA). Tungsten DCP-HTR probes were used during the measurements. The resistivity was estimated at five different points for each sample.

## 3. Results and Discussion

**Figure 1:**
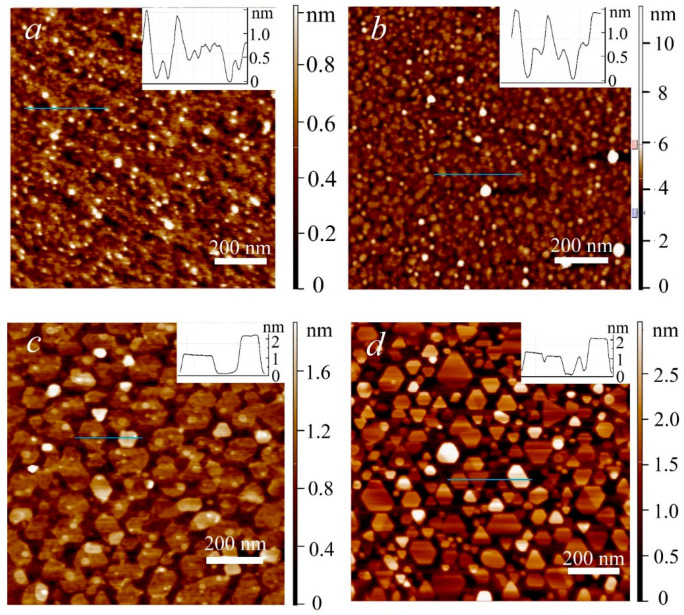
AFM image of MoS_2_ surface, samples deposited at 650 °C (**a**), 750 °C (**b**), 850 °C (**c**) and 950 °C (**d**).

In Figure 1 AFM images of MoS_2_ samples deposited at different temperatures are provided. The deposition time for these samples was 4 h at 650 °C, and 6 h at other temperatures. The images demonstrate that at 650 °C the substrate is completely covered with film, consisting of nanocrystal grains with a lateral size of 10–15 nm. With the deposition temperature increase to 750 °C, the grain size increases to 15–30 nm, resulting film is also almost continuous, with separate gaps. Qualitative changes occur at 850 °C: crystallite size sharply increases exceeding 100 nm. However, the crystallites are disconnected and do not form a continuous film. A further increase in temperature to 950 °C resulted in the formation of crystallites with a polygonal shape, mostly in the form of triangles with truncated vertices. The provided profiles demonstrate that the fine grains of the samples synthesized at lower temperatures are 1–2 monolayers thick, whereas the larger grains of the samples synthesized at higher temperatures are 2–3 monolayers thick.

The obtained relationship between growth temperature and grain size is in good agreement with that previously reported for MOCVD MoS_2_ processes without promoters. The low grain size and early grain coalescence at 650–750 °C are attributed to the high nucleation density. As the temperature rises, the diffusion rate increases resulting in the decrease of nucleation density. In particular, according to the data reported in [38], with the growth temperature increase from 670 °C to 955 °C, the nucleation density decreases from about 140 to about 15 (nuclei/µm^2^).

In order to estimate film continuity and grain size distribution, statistical processing of the AFM images in Figure 1c,d was carried out. The analysis suggests that at 850 °C approximately 65% of the substrate surface is covered with crystallites. At 950 °C, the continuity factor is reduced to about 55%. The average grain size at 850 °C is approximately 3900 nm^2^, while at 950 °C it is significantly lower constituting 1700 nm^2^. In Figure S1 a histogram of grain size distribution is provided, which demonstrates that the decrease in the average grain size at 950 °C occurs due to the fact that grains with a size of less than 2000 nm^2^ constitute a significantly larger fraction in this case. At 850 °C the distribution peak is approximately at 2000 nm^2^, in case of the samples deposited at 950 °C it is shifted to 50 nm^2^. Such an average grain size decrease due to the predominant formation of fine grains correlates with the previously reported data in the literature [30]. The maximum grain size at both temperatures constitutes about 1200 nm^2^.

The works published to date indicate that at temperatures above 900 °C, MoS_2_ decomposition significantly affects the growth process. In particular, in [38] the authors established that the domains formed at the seeding stage at 845 °C only increased in size slightly after the 6 h growth stage at 950 °C, while the average domain size decreased. In [39], with the increase of growth temperature to 850 °C in a single-stage process, the density and size of domains decreased significantly. The authors characterized this regime as desorption limited. Thus, at high temperatures, decomposition and desorption are the processes that hinder the film growth. In case of the present work, this may explain the decrease in the average size of crystalline domains at the 850 °C to 950 °C transition.

The surface morphology for continuous films of the second series, deposited at 650 °C and 950 °C was using SEM, the images are introduced in the Figure S2. The images comparison demonstrate that the maximum crystallite size slightly increased with deposition time increase. The homogeneous intergrain background in the images is formed by coalesced fine grains.

**Figure 2:**
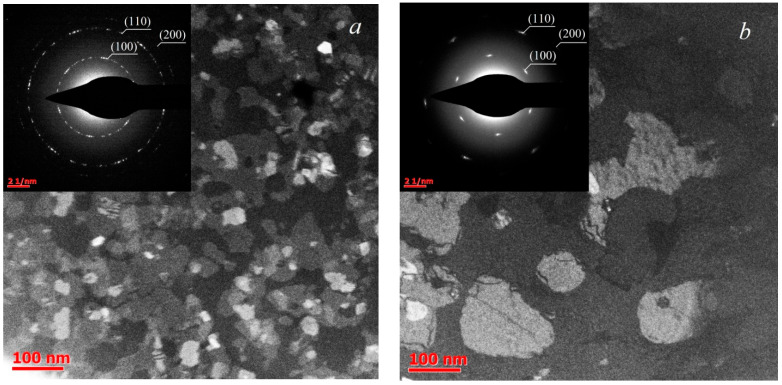
Plan-view TEM images of MoS_2_ films, deposited at 750 °C (**a**) and 850 °C (**b**). Insets show SAED patterns.

For structural TEM analysis, the samples synthesized at 750 °C and 850 °C were chosen, since according to the AFM data this temperature transition resulted in a dramatic morphology change. The deposition time of these samples was 6 h at 750 °C and 9 h at 850 °C. The resulting images are provided in Figure 2, with the corresponding SAED pictures in the insets. A 1 μm diaphragm was used in TEM analysis. The TEM results confirm that as the deposition temperature increases from 750 °C to 850 °C, the characteristic crystallite lateral size increases from 30–50 nm to approximately 100–150 nm. In addition, while the SAED pattern of the sample synthesized at 750 °C suggests a slightly preferred orientation of crystallites, the SAED pattern of the 850 °C sample demonstrates a clearly defined orientation. This indicates the epitaxial growth of MoS_2_ crystallites on the sapphire substrate. In this case, relative rotation angles of individual crystallites are multiples of 60°. In addition, it should be noted that the point reflections blurring indicates a misorientation within 4°.

**Figure 3:**
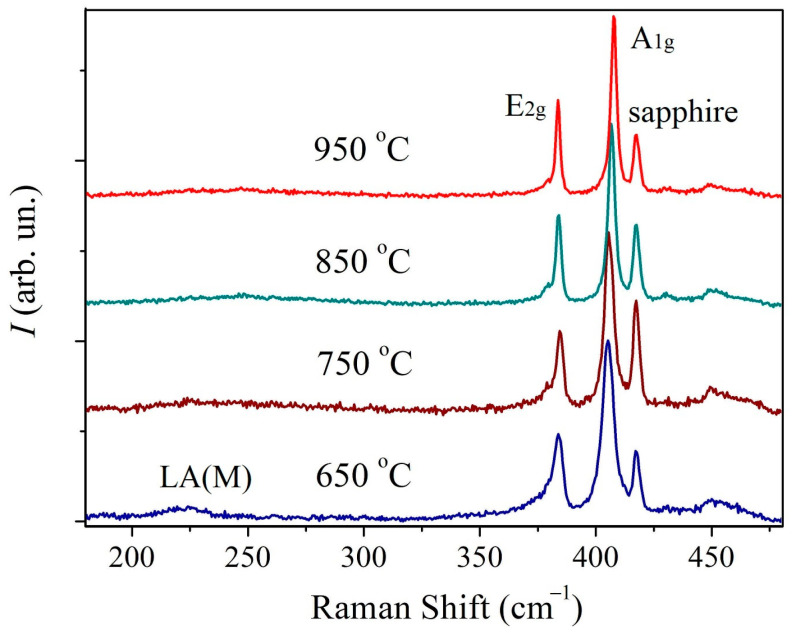
Raman spectra of MoS_2_ samples deposited at different temperatures.

In Figure 3 the Raman spectra of the first series of MoS_2_ samples are presented. The spectra contain first-order peaks denoted as E_2g_^1^ (Γ) and A_1g_ (Γ) corresponding to oscillatory modes inside the S-Mo-S layer in the parallel and perpendicular directions. The spectrum of the sample synthesized at 650 °C additionally demonstrates a broad LA(M) peak at about 227 cm^−1^ which is sensitive to lattice defects [42,43,44]. Previously, a correlation was established between the relative intensity of this peak and the density of both radiation-induced point defects [42], and grain boundaries [43,44]. This peak indicates a higher concentration of defects in this sample.

**Figure 4:**
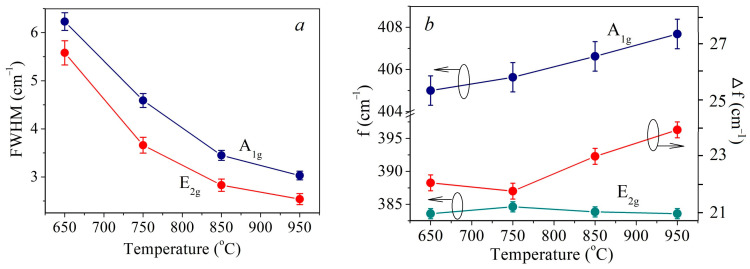
FMHM temperature dependency of E2g and A1g peaks (**a**), temperature dependency of E2g and A1g peaks position and their inter-shift (**b**).

In Figure 4a Full Width-Half Maximum (FWHM) temperature plot for the E_2g_^1^ and A_1g_ peaks is provided. For both peaks, the FWHM decreases monotonically with increasing temperature. A sharper decrease is observed in the temperature range 650–850 °C. In this range, the decrease in FWHM is presumably associated with an increase in the crystallite size. However, in the range of 850–950 °C, the average crystallite size decreases. Therefore, the persistence of the trend towards a FWHM decrease should be associated with an improvement in the crystal structure quality.

The temperature dependences of the E_2g_^1^ and A_1g_ peaks’ positions, as well as their inter-shift, are shown in Figure 4b. As the temperature increases, the E_2g_^1^ peak does not shift significantly and is situated at 383.6 cm^−1^. A_1g_ peak shifts monotonically towards the increasing wavenumber (blue-shift). This results in the peaks’ inter-shift increase from 21.4 cm^−1^ to 24.1 cm^−1^. It is known that the shift between the positions of these peaks for ultrathin MoS_2_ films depends on the number of monolayers and is often used to determine it [45,46,47]. For the investigated samples, such calculation suggests that the samples synthesized at 650 °C and 750 °C are 1–2 monolayers films, and the films fabricated at 850 °C and 950 °C are 2–3 monolayers thick. To confirm the thickness of the films additional AFM measurements were conducted measuring film step profiles. The thickness of the samples fabricated at 750 °C and 950 °C was determined as follows: film fragments were PMMA transferred from the sapphire substrate used in CVD onto the target SiO_2_/Si substrate using otherwise the same routine as for transfer on TEM grids. For the other two films obtained at 650 °C and 850 °C the thickness was estimated from the AFM profiles recorded over scratches. In Figure S3 the corresponding AFM images are provided. These results suggest that the films fabricated at 650 °C and 750 °C are 2 monolayers thick, whereas the films obtained at 850 °C and 950 °C consist of 3 monolayers. In case of the samples fabricated at 650–850 °C films the AFM and Raman spectroscopy data coincide. However, at 950 °C there is a discrepancy in film thickness as estimated from AFM and Raman data. According to the AFM profiles, the crystallites are 3 monolayers thick, whereas the Raman spectrum suggests that the film consists of 4 layers. In order to explain this discrepancy, it should be taken into account that, in addition to the number of monolayers, Raman spectra peak positions are affected by such factors as internal stresses in films [48], electron or hole doping [49], atomic structure defects, primarily sulfur vacancies (V_S_) as the most common defects in MoS_2_ [50]. In [50] the effect of the V_S_ concentration on the position of the E_2g_1 and A_1g_ peaks was demonstrated both experimentally and theoretically. At a V_S_ concentration within 1 at.%, the blue-shift of the A_1g_ peak may reach 1–2 cm^−1^, while the position of the E_2g_^1^ peak remains almost unchanged. However, at high V_S_ concentrations, E_2g_^1^ peak shift is more significant than that of the A_1g_ peak. Thus, the A_1g_ peak blue-shift by about 1 cm^−1^ with a temperature increase from 850 °C to 950 °C may be attributed to the V_S_ concentration increase of about 1 at.%.

**Figure 5:**
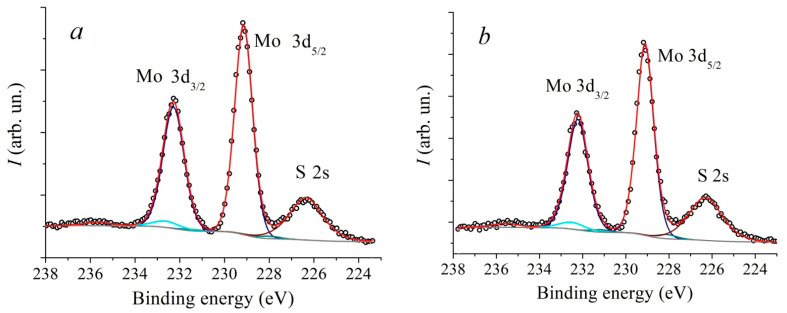

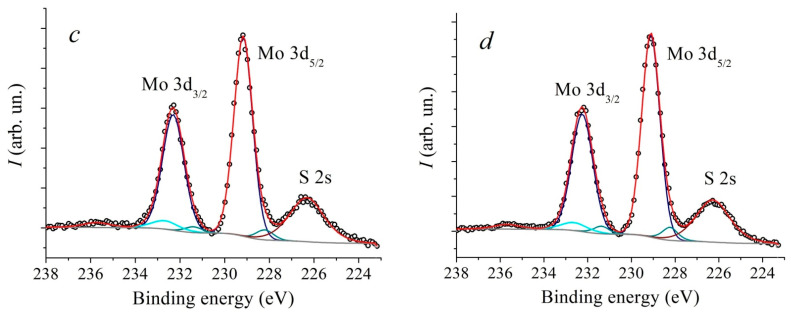
XPS core level Mo3d spectra of MoS_2_ samples, deposited at different temperatures: 650 °C (**a**), 750 °C (**b**), 850 °C (**c**) and 950 °C (**d**).

**Figure 6:**
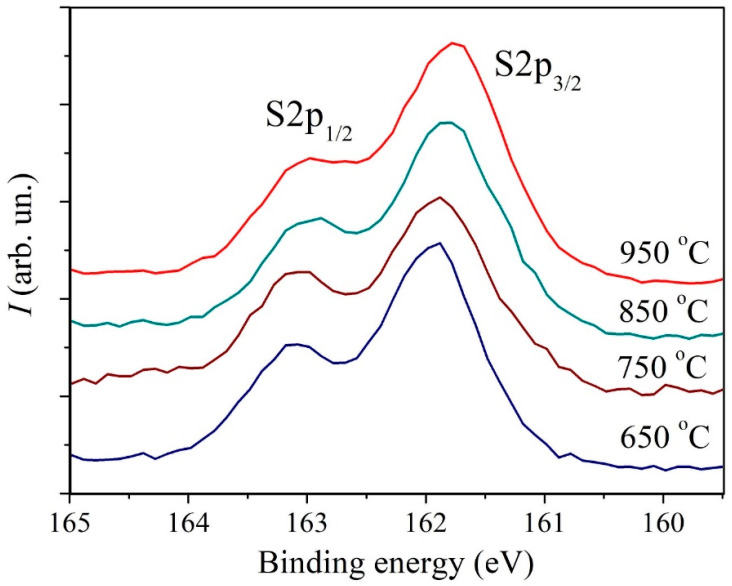
XPS core level S2p spectra of MoS_2_ samples deposited at different temperatures.

**Figure 7:**
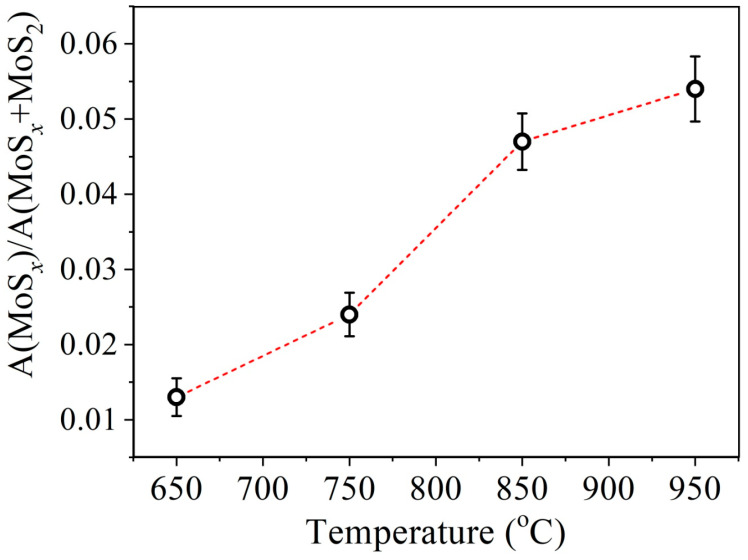
Temperature dependence of the relative concentration of non-stoichiometric MoS_x_ compound estimated from XPS spectra.

In Figure 5 and Figure 6 XPS spectra of Mo3d and S2p core levels of MoS_2_ samples synthesized at different temperatures are presented. The Mo3d spectra were decomposed into three doublets. The first doublet with the binding energy (BE) of Mo3d5/2 line of 229.1 eV and spin-orbit splitting of 3.15 eV corresponds to Mo^4+^ states in the 2H-MoS_2_ structure. The second doublet with BE = 232.5 eV corresponds to the Mo^6+^ state in the MoO_3_ compound. The third doublet with BE = 228.3 eV corresponds to the Mo state in the substoichiometric compound MoS_x_ [51,52,53]. The relative concentration of the Mo^6+^ state for all samples is approximately 5 at.%. At the same time, the concentration of the state corresponding to the MoS_x_ phase increases with an increase in the deposition temperature from approximately 1.3 at.% to 5.4 at.%. The temperature dependence plot of the relative concentration of non-stoichiometric MoS_x_ compound estimated from XPS spectra is provided in Figure 7. According to [51], the value BE = 228.3 eV for the MoS_x_ phase corresponds to the atomic concentrations ratio of [S]/[Mo] = 1.3. In the stoichiometric MoS_2_, each Mo atom is bonded to 6 S atoms. If the S:Mo ratio in the film is 1.3, each Mo atom lacks bonds with two S atoms on average. In order to not account for each missing S atom three times, the concentration V_S_ was calculated using the following formula: V_S_≈2Mo^x+^/(3· (Mo^x+^ + Mo^4+^))·100%, where Mo^x+^ is the MoS_x_ component intensity, and Mo^4+^ is MoS_2_ component intensity. The calculation carried out based on this data suggests that with an increase in the deposition temperature from 650 °C to 950 °C, the V_S_ concentration increases from about 0.9 at.% to 3.6 at.%. Direct calculation of x = [S]/[Mo] ratio provides a value of 2.0 ± 0.1 for all samples. However, using this calculation method, it is not possible to detect changes in the samples’ composition that are at the sensitivity limit of the XPS method. The S2p spectra also undergo changes with increasing temperature. In addition, a slight shift (by 0.1 eV) towards lower binding energy is observed.

**Figure 8:**
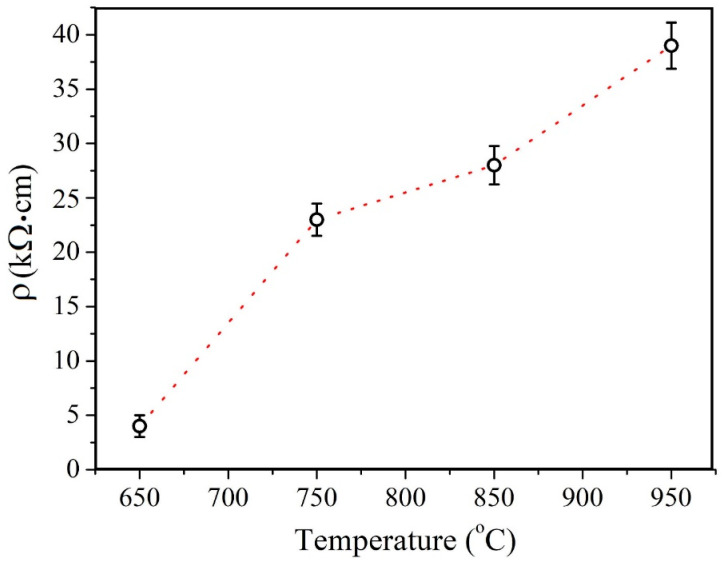
MoS_2_ films’ conductance temperature dependency.

In order to estimate the resistivity of the films, the sheet resistance was measured using the four-probe method at five different points for each sample. These measurements demonstrated a high degree of uniformity: the measured values remained within a 1% deviation. The resistance was estimated for continuous films fabricated in the second series. In Figure 8 a plot of the films’ resistivity versus the deposition temperature is provided. The results make it evident that the resistivity increases monotonically with the temperature increase.

The obtained results suggest that in films synthesized at lower temperatures consisting of relatively fine grains, more pronounced structural distortions are observed. They may be ascribed to both the high specific surface area of grain boundaries and the presence of point defects. This manifests in the broadening of the Raman spectra peaks. However, the Mo and S XPS spectra suggest that chemical states are more pure in this case. This means that the coalescence of grains may occur without the formation of a large number of vacancies. On the other hand, at a relatively high temperature, larger crystallites with a regular geometric shape and a more perfect structure are formed, which results in the narrowing of the Raman spectra peaks. However, the chemical states of the elements S and Mo turn out to be less pure due to the high concentration of V_S_. An analysis of published data shows that the typical concentration of V_S_ for MoS_2_ CVD films is 0.5–1 at.% [49,54]. There are two possible explanations for the increase in V_S_ concentration with increasing growth temperature. First is that the desorption rate increases with temperature for sulfur more rapidly than for molybdenum. The second is that the concentration of V_S_ in the synthesized MoS_2_ films is proportional to the density of grain boundaries. In [55] authors employed DFT calculations to demonstrate thatV_S_ have lower formation energy at grain boundaries S-polarized defect complexes (5|7) at the grain boundaries are readily transformed into (4|6) complexes with double sulfur vacancies (V_2S_). This also explains the fact that in MoS_2_ CVD films the V_S_ concentration is usually several orders of magnitude higher than in exfoliated flakes. In addition, the second explanation is supported by the fact that the concentration of V_S_ increases non-monotonically with temperature increase, it demonstrates a significant increase with temperature transition from 750 °C to 850 °C. This also correlates with grain size variation. For the crystallites with the lateral size below 10 nm, their coalescence does not result in high V_S_ concentration due to disruption of the interatomic bonds.

In terms of electrical properties it should be pointed out that as was shown in [54] an increase in the V_S_ concentration to 3% reduces the mobility of charge carriers by two orders of magnitude. In addition, the intergrain boundaries are a crucial factor in polycrystalline TMDC films conductivity [56,57,58]. In [34] the significant resistance increase in magnetron deposited MoS_2_ film, annealed at high temperatures, is reported. The sheet resistance of resulting films is reported to be as high as 8 MΩ/□ for 50 nm film annealed at 900 °C. The authors suggest that this increase is caused by grain boundaries’ formation, accompanying grain crystallization. We argue that the resistivity of the films under investigation is primarily governed by a high concentration of vacancies at the crystallite interfaces, which increases the charge carriers scattering.

## 4. Conclusions

In this work, the structure, chemical states, and electrical properties of MOCVD synthesized MoS_2_ films were studied at various temperatures from 650 °C to 950 °C. In the temperature range of 650 °C to 850 °C, an increase in the average size of crystallites with increasing temperature is observed. With further deposition temperature increase, at 950 °C, the crystallites’ size distribution peak shifts toward lower values and the average size decreases. The advantages of a high deposition temperature should be more pronounced if parasitic nucleation is suppressed at later growth stages. It has also has been demonstrated that epitaxial growth on a sapphire substrate occurs as the temperature reaches 850 °C. In addition, the opposite behavior of the Raman and XPS spectra is revealed: peak narrowing and broadening, respectively. The narrowing of the Raman spectra reflects the improvement in the crystal structure, while the broadening of the XPS spectra reflects an increase in the concentration of V_S_. The calculation based on the XPS spectra showed an increase in the V_S_ concentration from about 0.9 to 3.6 at.%. The electrical resistivity of the films increased monotonically from 4 kΩ·cm at 650 °C to 39 kΩ·cm at 950 °C. This may be attributed to an increase in the V_S_ concentration at the crystallite interfaces.

## Data Availability

Any additional data supporting the findings are available from the corresponding author upon reasonable request.

## Supplementary Materials

The following supporting information can be downloaded at: https://www.mdpi.com/article/10.3390/nano13192712/s1, Figure S1: Crystallite size distribution histogram for MoS_2_ samples deposited at 850 °C and 950 °C; Figure S2: SEM-images of MoS_2_ films’ surface, deposited at 950 °C for 9 h (a) and at 650 °C for 6 h (b). The brighter regions correspond to thicker film grains, darker background to the thinner underlying film; Figure S3: AFM-images and step profiles along the white lines (insets) for films deposited at 650 °C (a), 750 °C (b), 850 °C (c), and 950 °C (d).
