# Supplementary material for: Temperature-Dependent Structural and Electrical Properties of Metal-Organic CVD MoS2 Films
Source: Nanomaterials (Basel). 2023 Oct 6;13(19):2712. doi: 10.3390/nano13192712 (PMC10574732; doi:10.3390/nano13192712)
Supplement: Supplementary file 1 [file nanomaterials-13-02712-s001.zip › nanomaterials-2599993-supplementary.pdf]

nanomaterials
MDPI

Supporting Information

Temperature-Dependent Structural and Electrical Properties of Metal-Organic CVD MoS_2 Films

Roman I. Romanov [1], Ivan V. Zabrosaev [1], Anastasia A. Chouprik [1], Dmitry I. Yakubovsky [2], Mikhail K. Tatmyshevskiy [2], Valentyn S. Volkov [2] and Andrey M. Markeev [1,]*

1 Center of Shared Research Facilities, Moscow Institute of Physics and Technology (National Research University), Dolgoprudny 141701,Russia; romanov.ri@mipt.ru (R.I.R.); zabrosaev@phystech.edu (I.V.Z.); chouprik.aa@mipt.ru (A.A.C.)
2 Center for Photonics & 2D Materials, Moscow Institute of Physics and Technology (National Research University), Dolgoprudny 141700, Russia dmitrii.yakubovskii@phystech.edu (D.I.Y.); mikhail.tatmyshevskiy@phystech.edu (M.K.T.); volkov.vs@mipt.ru (V.S.V.)
* Correspondence: markeev.am@mipt.ru; Tel.: +7-(919)-411-6809

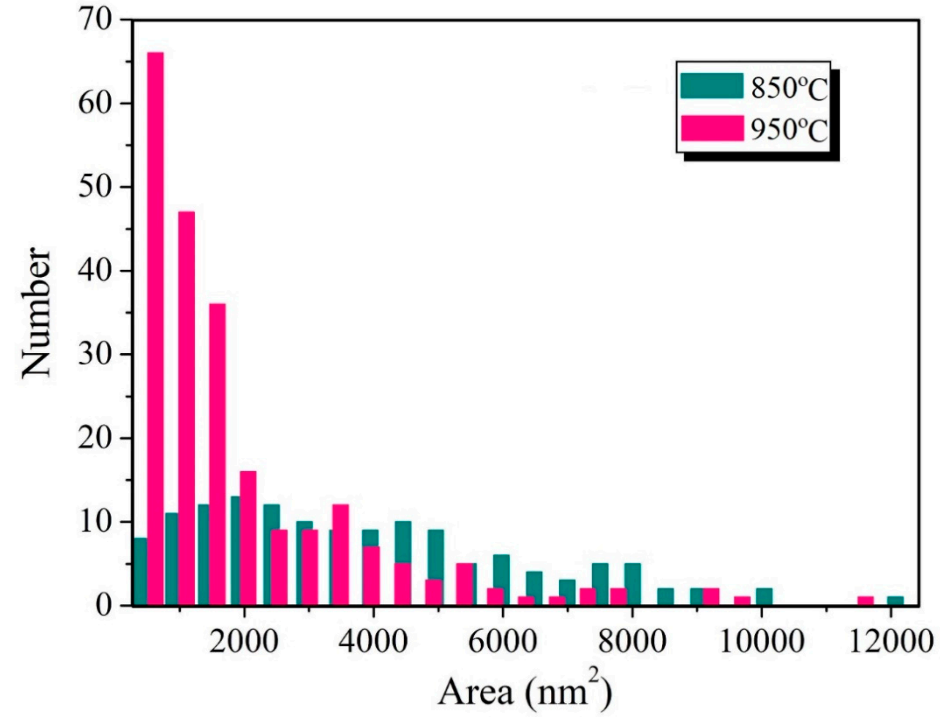

Figure S1. Crystallite size distribution histogram for MoS_2 samples deposited at 850 °C and 950 °C.

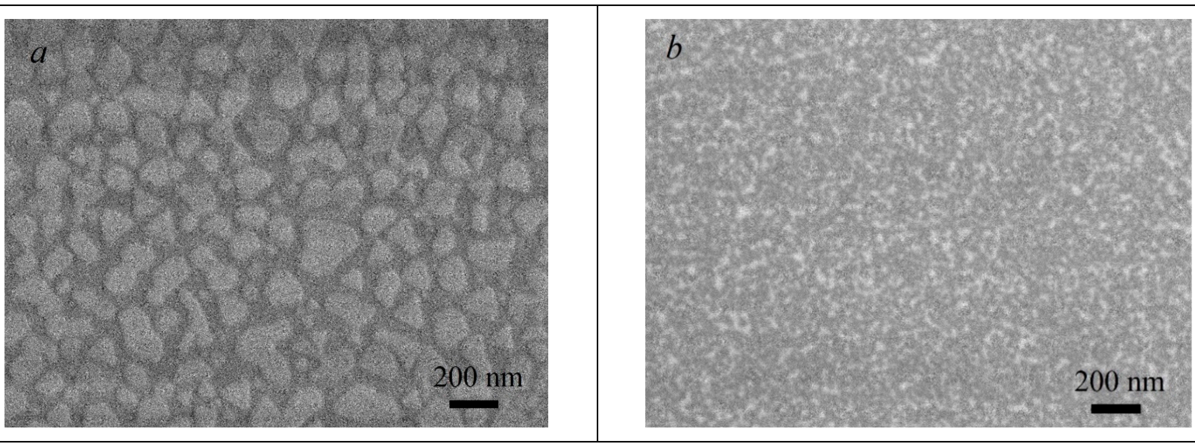

Figure S2- SEM-images of MoS_2 films' surface, deposited at 950 °C for 9 hours (**a**) and at 650 °C for 6 hours (**b**). The brighter regions correspond to thicker film grains, darker background to the thinner underlying film.

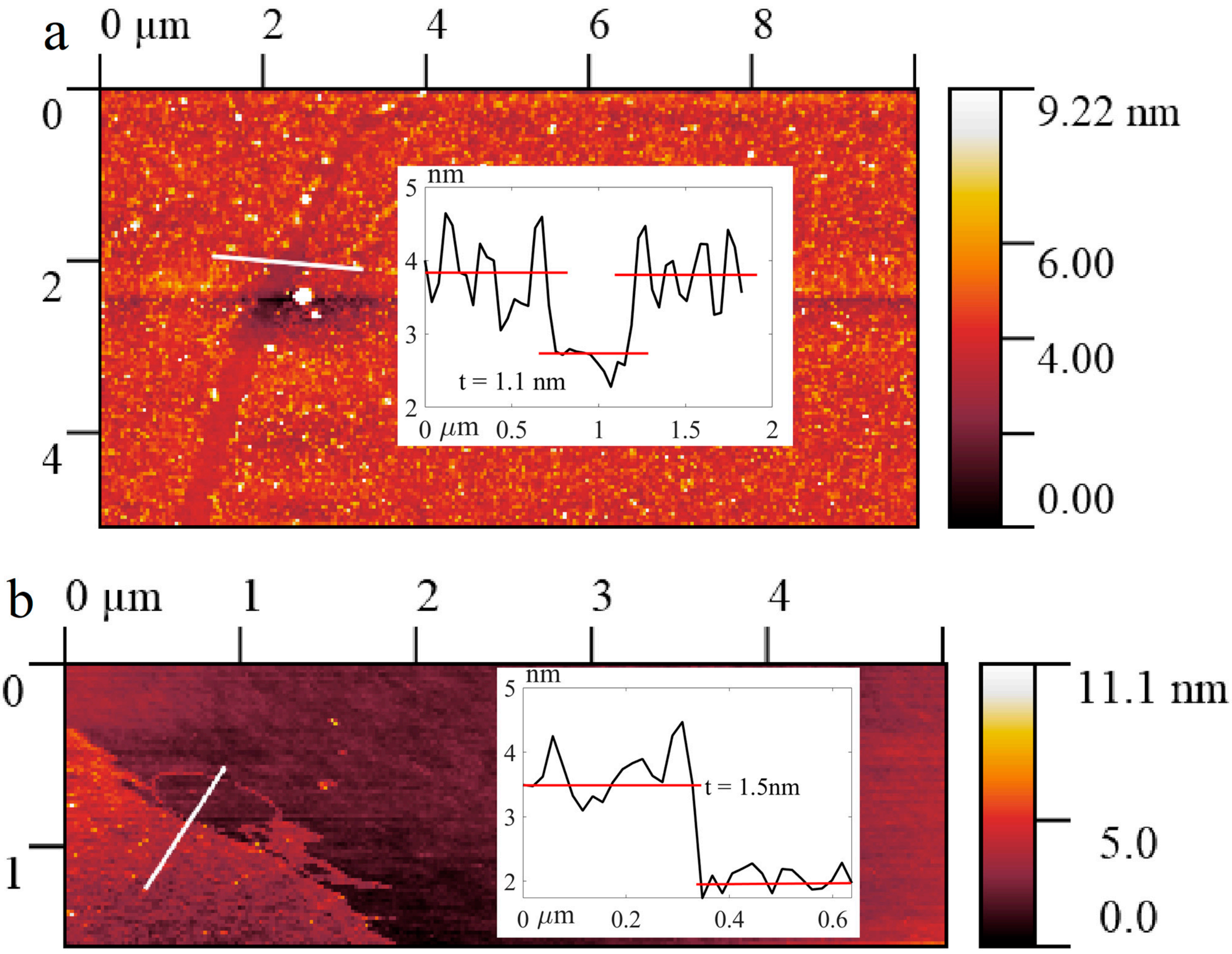

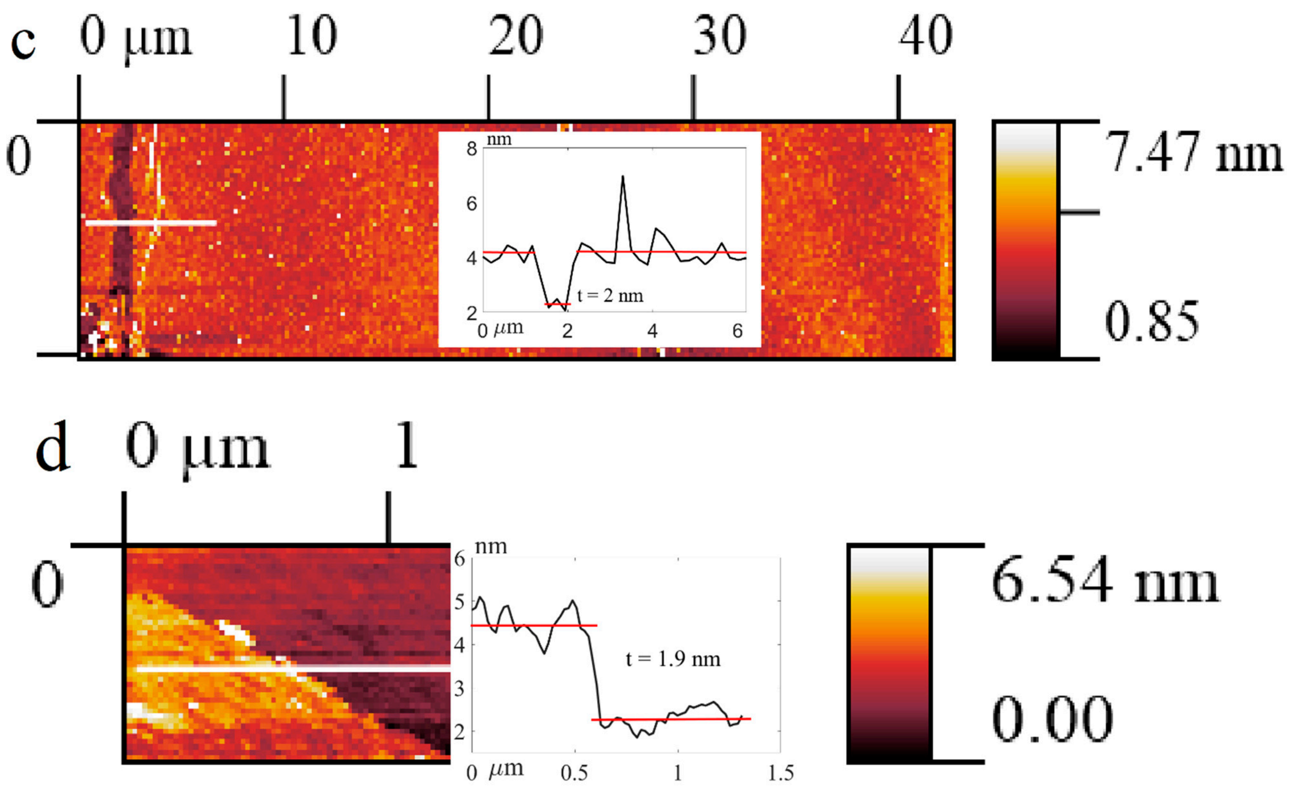

Figure S3- AFM-images and step profiles along the white lines (insets) for films deposited at 650 °C (**a**), 750 °C (**b**), 850 °C (**c**), and 950 °C (**d**).
